# Long-Term Continuous Corticosterone Treatment Decreases VEGF Receptor-2 Expression in Frontal Cortex

**DOI:** 10.1371/journal.pone.0020198

**Published:** 2011-05-27

**Authors:** Kristy R. Howell, Ammar Kutiyanawalla, Anilkumar Pillai

**Affiliations:** 1 Department of Psychiatry and Health Behavior, Georgia Health Sciences University, Augusta, Georgia, United States of America; 2 Medical Research Service, Charlie Norwood Veterans Affairs Medical Center, Augusta, Georgia, United States of America; University of Queensland, Australia

## Abstract

**Objective:**

Stress and increased glucocorticoid levels are associated with many neuropsychiatric disorders including schizophrenia and depression. Recently, the role of vascular endothelial factor receptor-2 (VEGFR2/Flk1) signaling has been implicated in stress-mediated neuroplasticity. However, the mechanism of regulation of VEGF/Flk1 signaling under long-term continuous glucocorticoid exposure has not been elucidated.

**Material and Methods:**

We examined the possible effects of long-term continuous glucocorticoid exposure on VEGF/Flk1 signaling in cultured cortical neurons *in vitro*, mouse frontal cortex *in vivo*, and in post mortem human prefrontal cortex of both control and schizophrenia subjects.

**Results:**

We found that long-term continuous exposure to corticosterone (CORT, a natural glucocorticoid) reduced Flk1 protein levels both *in vitro* and *in vivo*. CORT treatment resulted in alterations in signaling molecules downstream to Flk1 such as PTEN, Akt and mTOR. We demonstrated that CORT-induced changes in Flk1 levels are mediated through glucocorticoid receptor (GR) and calcium. A significant reduction in Flk1-GR interaction was observed following CORT exposure. Interestingly, VEGF levels were increased in cortex, but decreased in serum following CORT treatment. Moreover, significant reductions in Flk1 and GR protein levels were found in postmortem prefrontal cortex samples from schizophrenia subjects.

**Conclusions:**

The alterations in VEGF/Flk1 signaling following long-term continuous CORT exposure represents a molecular mechanism of the neurobiological effects of chronic stress.

## Introduction

Stress and elevated glucocorticoid levels are known to be associated with a number of neuropsychiatric disorders including depression and schizophrenia. Although acute treatment with corticosteroid is shown to be neuroprotective [1] their chronic effects are deleterious to the structural and functional plasticity of adult brain [2]. Moreover, many of the effects of chronic stress are thought to be mediated by stress-induced increases in circulating levels of glucocorticoids [3], [4]. Animal studies have shown a number of behavioral abnormalities similar to depression and anxiety following chronic administration of glucocorticoids [5]–[7]. However, the mechanism underlying long-term continuous glucocorticoid-induced alterations in neuroplasticity is still not clear.

Vascular endothelial growth factor (VEGF) is a neuroprotective, angiogenic and neurotrophic molecule [8]. VEGF is known to mediate its biological functions via activation of the protein tyrosine kinase receptors, VEGF receptor 1 (VEGFR-1/Flt1) and VEGFR-2 (KDR/Flk1) [9]. VEGF and its receptors are expressed on neurons and astrocytes, and VEGF induces neuronal outgrowth. Flk1 has been shown to mediate VEGF action in neuronal functions [10], [11] and activation of Flk1 allows the receptors to associate with various downstream effector molecules including phosphatidylinositol 3-kinase (PI3K) [12], [13]. PI3K/Akt signal transduction pathway has been identified as an important mediator VEGF signaling downstream of Flk1 [14].

The prefrontal cortex (PFC) is a brain region involved in higher-order cognitive and affective processing, as well as executive function [15], [16]. The PFC is also a target region for glucocorticoid effects, as it has a rich population of glucocorticoid receptors [17], [18]. High doses of glucocorticoids are known to impair PFC-dependent working memory in rodents [19], [20] and humans [21].Chronic corticosterone (CORT) treatment has also been shown to produce neuronal impairment in the PFC including the remodeling of pyramidal neurons, significantly reduced distal dendritic spines of neurons and neuronal loss [22]–[24]. VEGF signaling is known to play an important role in cognitive functions and neuroprotection [10], [11]. A recent study has shown that pharmacological inhibition of Flk1 signaling can block the behavioral actions of fluoxetine in rats subjected to chronic stress [25] indicating a possible role of Flk1 signaling in stress-mediated behavioral changes. However, the effects of chronic glucocorticoid on VEGF signaling remain unknown. Specifically it is not known whether long-term continuous glucocorticoid exposure can cause alterations in VEGF signaling pathway in PFC. Studies on the effect of glucocorticoids on VEGF signaling might provide valuable information on the molecular mechanism of the neurobiological effects of chronic glucocorticoid exposure.

In the present article, we investigated the effects of long-term continuous CORT exposure on VEGF/Flk1 signaling in cultured cortical neurons *in vitro*, mouse frontal cortex *in vivo*, and in post mortem human prefrontal cortex of both control and schizophrenia subjects.

## Results

### Long-term Continuous CORT Treatment Does Not Change Neuronal Cell Viability

Neuronal cell viability was measured at 48, 72 and 96 h exposure of cortical neurons by MTT assay. We did not find any significant change in neuronal cell viability at any of the treatment point in CORT (I µM) treated cells as compared to vehicle treated cells (data not shown).

### Long-term Continuous CORT Treatment Decreases Flk1 Protein Levels *in vitro* and *in vivo*

**Figure 1:**
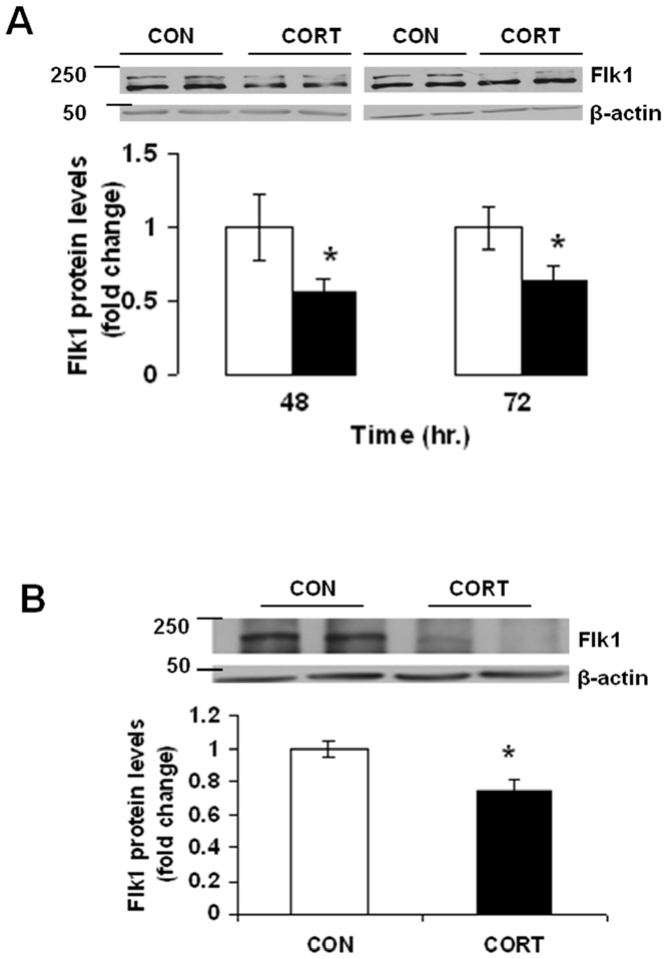
Long-term Continuous CORT treatment decreases Flk1 protein levels *in vitro* and *in vivo*. (*A*) CORT (CORT; I µM) was applied to mouse primary cortical neurons at DIV 5. Flk1 protein levels were determined by western blotting analysis at 48 hand 72 h following CORT treatment. CON means DMSO treatment. Data represent mean±SE. (*n* = 6) expressed as fold change in Flk1 protein levels as compared to CON. β-actin is the loading control. *P<0.05 (Bonferroni's test). (*B*) Flk1 protein levels in frontal cortex of mice treated with CORT or vehicle control (CON; 0.45% hydroxypropyl-β-cyclodextrin) for 7 weeks. Data represent mean±SE (*n* = 6–8) expressed as fold change in Flk1 protein levels as compared to CON. *P<0.01 (t test).

Expression of Flk1 was examined at 12, 24, 48 and 72 h exposure of primary cortical neurons to CORT or vehicle by western blot analysis using Flk1 antibody. The densitometric values for Flk1 were corrected for β-actin. No change in Flk1 protein levels was found at 12 and 24 h following CORT treatment (data not shown). In 24 and 48 h treatment groups, data from two-way ANOVA revealed a significant time x treatment interaction [F(1, 20) = 19.27, p<0.01], but no significant main effect of time [F(1,20) = 0.382, N.S.] or treatment [F(1, 20) = 0.555, N.S.]. Post hoc analysis by Bonferroni's Multiple Comparison test showed a significant decrease in Flk1 protein levels at 48 h (t = 4.112, p<0.05), which lasted up to at least 72 h (t = 3.977, p<0.05) (Fig. 1*A*). Next, we examined Flk1 protein levels in mice treated with CORT for 7 weeks. Western blot analysis showed a significant decrease in Flk1 protein levels in frontal cortex of mice treated with CORT for 7 weeks as compared to vehicle-treated mice (Fig. 1*B* *;* t = 3.323, df = 10, p = 0.0039). In addition, we found a significant decrease in serum Flk1 protein levels in mice treated with CORT for 7 weeks (113.18±9.55 ng/mL vs 78.63±5.77 ng/mL (mean±SE); t = 2.702, df = 8, p = 0.035).

### Long-term Continuous CORT Treatment Alters p-PTEN, p-Akt and p-mTOR Levels in Cortical Neurons

**Figure 2:**
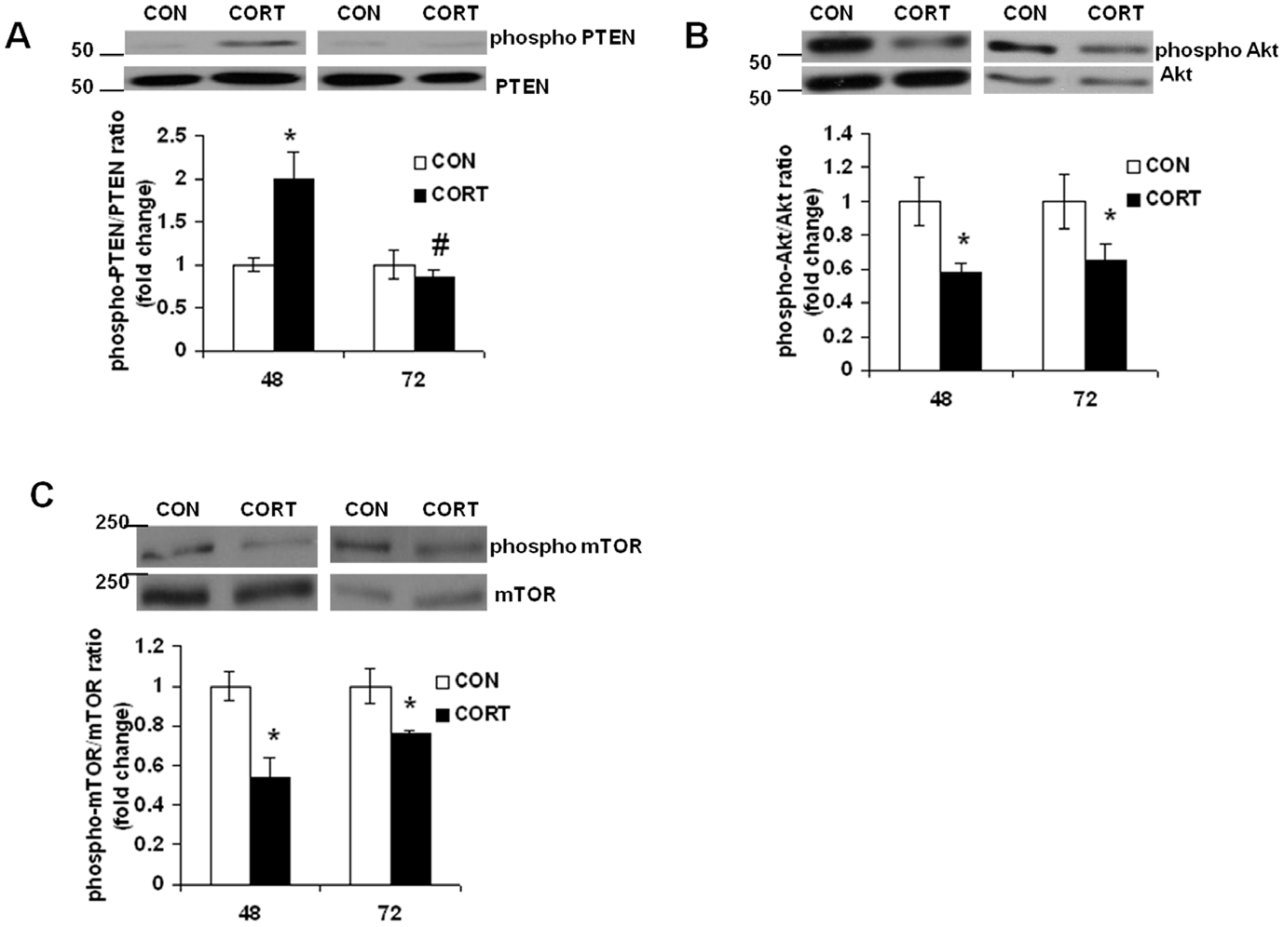
Long-term Continuous CORT treatment alters phospho PTEN, phospho Akt and phospho mTOR protein levels in cortical neurons. CORT (CORT; I µM) was applied to mouse primary cortical neurons at DIV 5. Cell lysates collected at 48 h or 72 h following CORT treatment were used for western blot analysis. CON means DMSO treatment. Data represent mean±SE (*n* = 6) expressed as fold change in *(A)* phospho PTEN to total PTEN ratio, *(B)* phospho Akt to total Akt ratio and *(C)* phospho mTOR to total mTOR ratio as compared to CON. *P<0.01 versus CON; #*P*<0.01 versus values at 48 h (Bonferroni's test).

Several studies have reported PTEN/Akt/mTOR pathway as a key downstream signaling to Flk1 [14]. To determine whether the changes in Flk1 protein levels result in alterations in its downstream signaling pathway, the PTEN/Akt/mTOR pathway was investigated. Cortical neurons were exposed to CORT for 48 or 72 h, and equal amounts of lysates were separated by PAGE and immunoblotted using phosphospecific PTEN antibody. The two-way ANOVA revealed significant effects of treatment [F(1, 20)  = 5.7, p<0.05], time [F(1, 20) = 5.77, p<0.01] and time x treatment interaction [F(1,20) = 9.246, p<0.05]. Subsequent comparisons by Bonferroni's Multiple Comparison test indicated that CORT significantly stimulated the phosphorylation of PTEN at 48 h of exposure compared with the control group (Fig. 2A; t = 3.838, p<0.05). However, a significant reduction in phospho-PTEN levels were found at 72 h as compared to levels at 48 h (t = 4.833, p*<*0.01), but no change as compared to vehicle-treated cells. Next, we examined the effect of CORT treatment on phospho-Akt levels in neurons. Data from two-way ANOVA showed a significant effect of treatment [F(1, 20) = 30.43, p<0.001], but no time x treatment interaction [F(1, 20) = 0.353, N.S.] or main effect of time [F(1,20) = 0.002, N.S.]. Post hoc analysis showed a significant reduction in phospho-Akt levels at 48 h following CORT treatment as measured by phosphorylation of serine 473 (Fig. 2*B*; t = 4.472, p<0.01). Also, a significant reduction in phospho Akt levels was observed when examined at 72 h following CORT treatment (t = 3.37, p<0.05). Since mTOR has a critical function in transducing signals from PI3K/Akt cascade, we next investigated the activation status of mTOR in neurons following CORT treatment. We found significant effects of treatment [F(1, 20) = 35.84, p<0.001], time [F(1,20) = 5.774, p<0.05] and time x treatment interaction [F(1, 20) = 6.786, p<0.05]. Post hoc analysis showed a significant reduction in phospho-mTOR levels at 48 h (t = 7.238, p<0.001) and 72 h (t = 3.581, p<0.05) following CORT exposure (Fig. 2*C*).

### Effects of Long-term Continuous CORT Treatment on VEGF Protein Levels *in vitro* and *in vivo*

**Figure 3:**
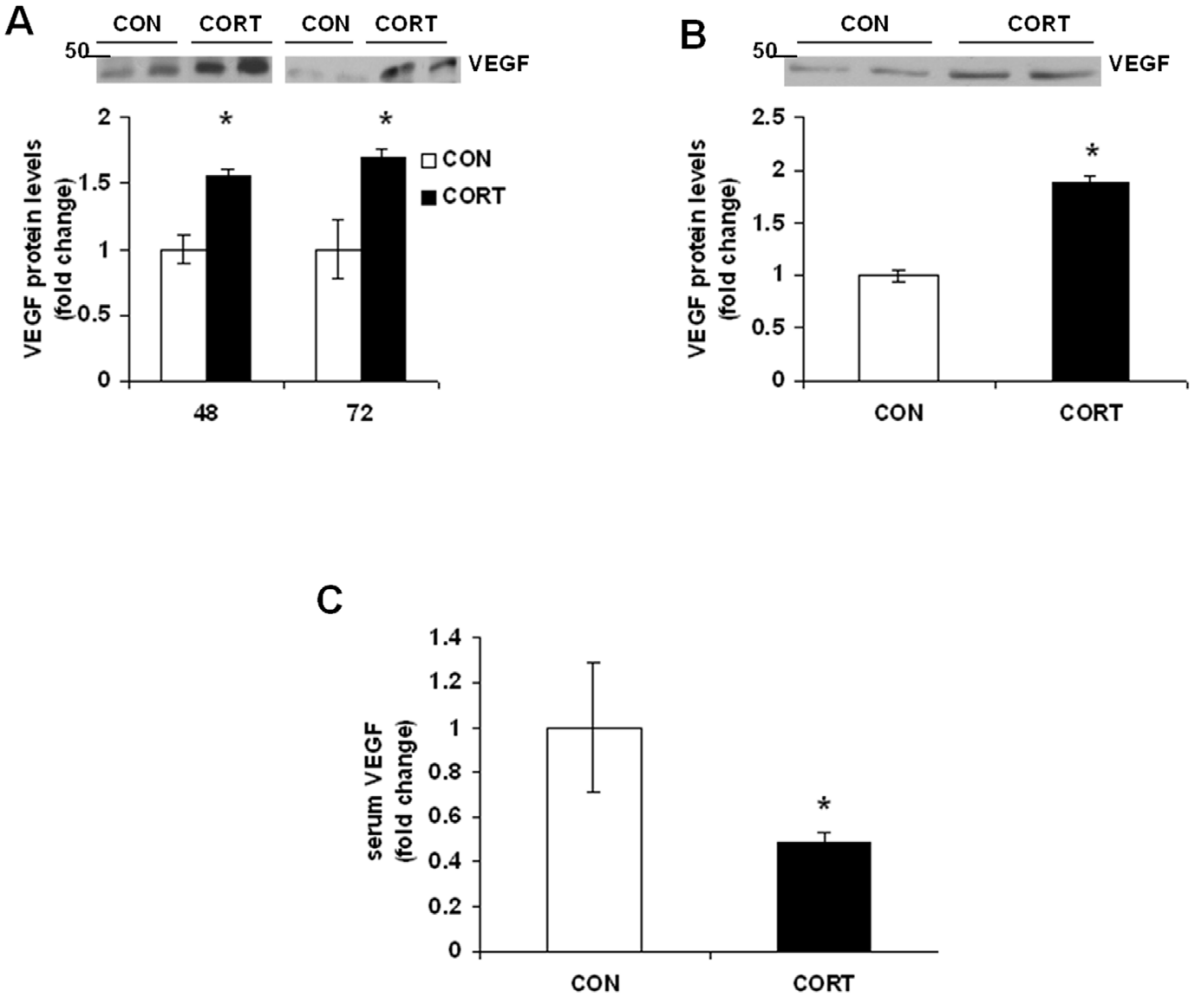
Long-term Continuous CORT treatment increases VEGF protein levels *in vitro* and *in vivo*. (*A*) CORT (CORT; 1 µM) was applied to mouse primary cortical neurons at DIV 5. VEGF protein levels were determined by western blotting analysis at 48 hand 72 h following CORT treatment. CON means DMSO treatment. Data represent mean±SE (*n* = 6) expressed as fold change in VEGF protein levels as compared to CON. *P<0.05 (Bonferroni's test). (*B*) VEGF protein levels in frontal cortex of mice treated with CORT (5 mg/kg) or vehicle control (CON; 0.45% hydroxypropyl-β-cyclodextrin) for 7 weeks were determined by western blot analysis. Data represent mean±SE (*n* = 5) expressed as fold change in VEGF protein levels as compared to CON. *P<0.01 (t test). *(C)* VEGF protein levels in serum samples collected from mice treated with CORT (CORT; 5 mg/kg) or vehicle control (CON; 0.45% hydroxypropyl-β-cyclodextrin) for 7 weeks were analysed by ELISA. Data represent mean±SE (*n* = 5–6) expressed as fold change in VEGF protein levels as compared to CON. *P<0.01 (t test).

To examine whether CORT treatment alters VEGF expression in cortex, we analysed VEGF protein levels in primary cortical neurons as well as in frontal cortex samples from mice treated with CORT for 7 weeks. In 24 and 48 h treatment groups, data from two-way ANOVA revealed a significant time x treatment interaction [F(1, 20) = 28.37, p<0.001], main effect of time [F(1,20) = 52.42, p<0.001] and treatment [F(1, 20) = 21.656, p<0.01]. Post hoc analysis showed that VEGF level was dramatically increased by nearly 56% when examined at 48 h of CORT exposure (Fig. 3A; t = 4.676, p<0.05), which lasted up to at 72 h (t = 3.856, p<0.05). We found a significant increase in VEGF protein levels in frontal cortex of mice treated with CORT for 7 weeks as compared to vehicle-treated mice (Fig. 3*B*; t = 10.04, df = 8, p<0.0001). In addition, we have examined VEGF levels in serum samples collected from mice treated with vehicle or CORT for 7 weeks. We found a significant decrease in serum VEGF levels following CORT treatment (Fig. 3C; t = 2.39, df = 9, p = 0.04).

### PI3K Signaling Is Not Involved in Long-term Continuous CORT-induced Increases in VEGF Protein Levels

**Figure 4:**
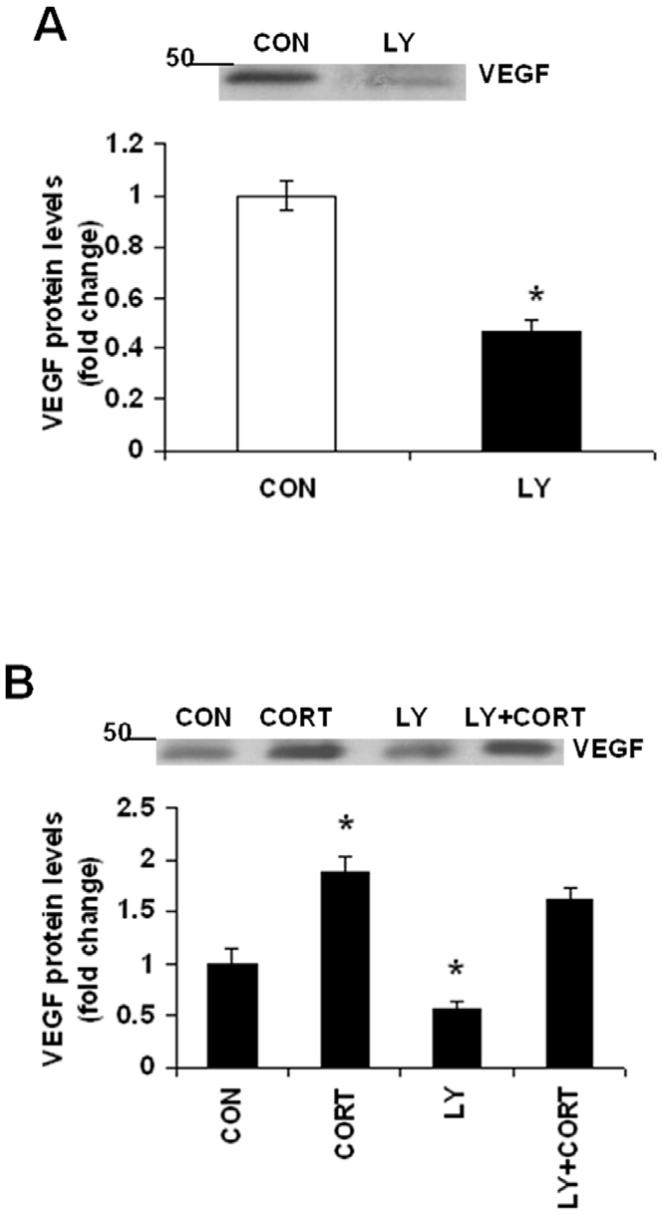
PI3K signaling is not involved in long-term continuous CORT-induced increases in VEGF protein levels. (*A*) VEGF protein levels were determined by western blotting analysis in neuronal cell lysates treated with LY294002 (LY; 20 µM) for 48 h. CON means DMSO treatment. Data represent mean±SE (*n* = 6) expressed as fold change in VEGF protein levels as compared to CON. *P<0.05 (Bonferroni's test). (*B*) Pretreatment with LY did not prevent CORT-induced induction in VEGF protein levels in neurons. Cortical neurons at DIV 5 were treated with LY (20 µM) for 30 min followed by CORT (1 µM) exposure for 48 h. VEGF protein levels were determined in cell lysates by western blot analysis. CON means DMSO treatment. Data represent mean±SE (*n* = 6) expressed as fold change in VEGF protein levels as compared to CON. *P<0.05 (Bonferroni's test).

Since PI3K signaling is also involved in the regulation of VEGF expression, we examined whether the changes in the above signaling molecules observed following CORT exposure are upstream to VEGF. In the first set of experiments, we determined the role of PI3K signaling in the regulation of VEGF expression. Cortical neurons were treated with PI3K inhibitor, LY294002 (LY) and VEGF protein levels were determined by immunoblotting analysis. We found a significant reduction in VEGF protein levels in neurons treated with LY for 48 h indicating a possible role of PI3K in VEGF regulation (Fig. 4*A* *;* t = 6.975; df = 10, p = 0.002). Next, we examined whether LY294002 can attenuate CORT-induced increases in VEGF protein levels. LY failed to inhibit CORT-induced increase in VEGF levels in neurons (Fig. 4*B*) indicating pathways other than PI3K might be involved in CORT-induced VEGF regulation.

### Long-term Continuous CORT-induced Flk1 Regulation Is Mediated Through Calcium

**Figure 5:**
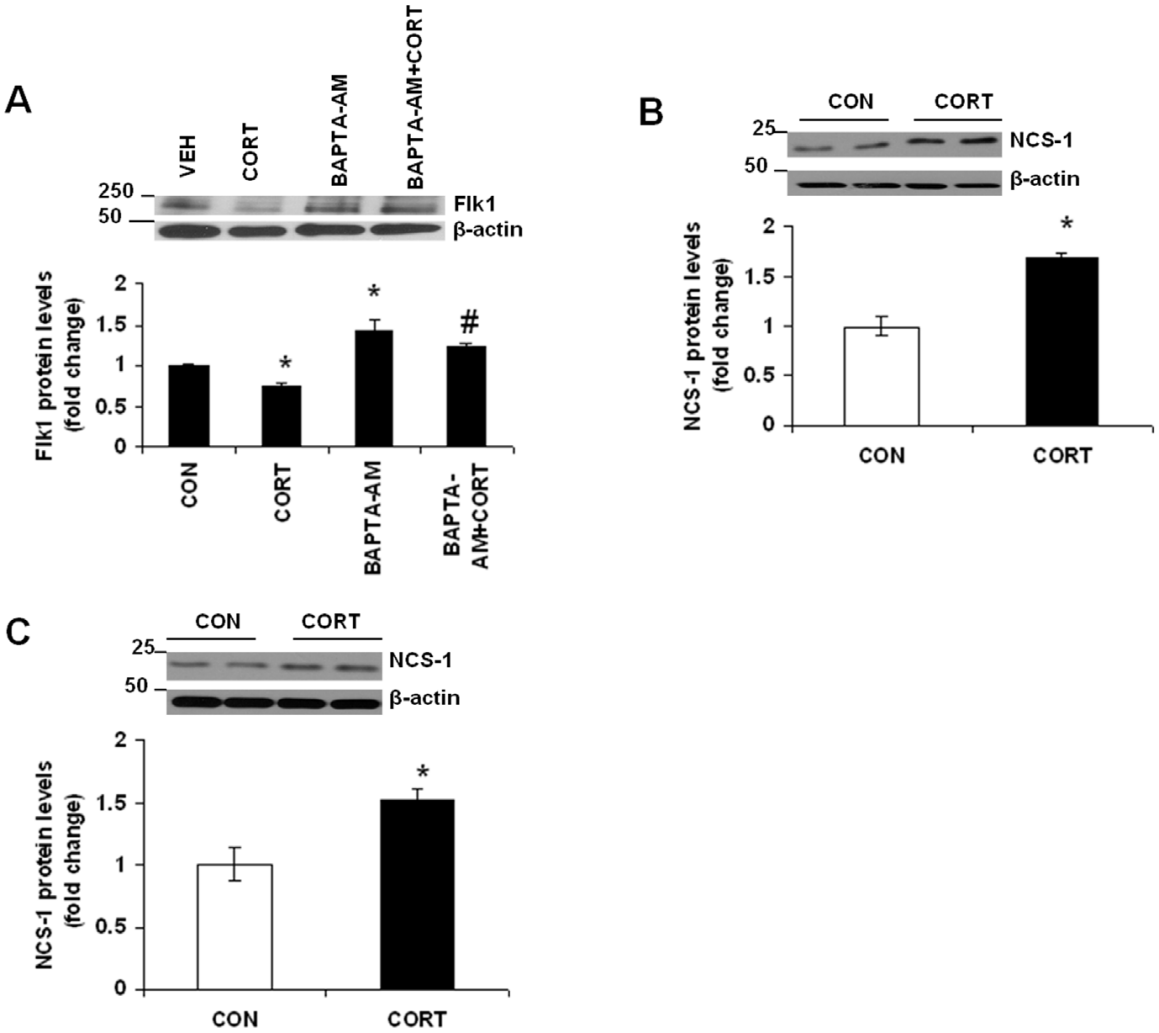
Chronic CORT-induced Flk1 regulation is mediated through calcium. (*A*) Calcium chelator BAPTA-AM blocked CORT (CORT)-induced reduction in Flk1 protein levels. BAPTA-AM (50 µM) was applied 30 min before CORT (1 µM) treatment to cultured neurons at DIV 5. Cell lysates were collected at 48 h after CORT treatment and Flk1 protein levels were determined by western blot analysis. CON means DMSO treatment. Data represent mean±SE (*n* = 5) expressed as fold change in Flk1 protein levels as compared to CON. *P<0.01 versus CON; #*P*<0.01 versus CORT (Bonferroni's test). (*B*) Chronic CORT treatment increases NCS-1 protein levels in neurons. CORT (CORT; 1 µM) was applied to mouse primary cortical neurons at DIV 5. NCS-1 protein levels were determined by western blotting analysis at 48 h following CORT treatment. CON means DMSO treatment. Data represent mean±SE (*n* = 5) expressed as fold change in NCS-1 protein levels as compared to CON. *P<0.01 (Bonferroni's test). (C) Chronic CORT treatment increases NCS-1 protein levels in mouse frontal cortex. NCS-1 protein levels in frontal cortex of mice treated with CORT (5 mg/kg) or vehicle control (CON; 0.45% hydroxypropyl-β-cyclodextrin) for 7 weeks were determined by western blot analysis. Data represent mean±SE (*n* = 6) expressed as fold change in NCS-1 protein levels as compared to CON. β-actin is the loading control.*P<0.05 (Bonferroni's test).

CORT treatment can regulate concentration of intracellular calcium ions (Ca^2+^) by modulating extracellular Ca^2+^ influx and intracellular Ca^2+^ pools [31]–[33]. To assess whether Ca^2+^ contributed to Flk1 regulation by chronic CORT exposure, we treated neurons with the membrane-permeable chelator BAPTA-AM. Application of BAPTA-AM abolished the decrease in Flk1 protein levels observed in the presence of CORT alone (Fig. 5*A* *;* F(3, 16) = 19.02; p<0.01). In addition, a significant increase in Flk1 expression was found in cells treated with BAPTA-AM alone as compared to vehicle-treated cells (p<0.05). The role of calcium in mediating CORT effects on Flk1 protein levels was further studied by examining the protein levels of neuronal calcium sensor-1 (NCS-1) in primary cortical neurons as well as in mouse frontal cortex following CORT exposure. NCS-1 is the mammalian ortholog of frequenin, a calcium-binding protein implicated in mediating several aspects of neurotransmission, including ion channel regulation [34], [35] and neurotransmitter release [36]–[38]. We found a significant increase in NCS-1 protein levels in cortical neurons treated with CORT for 48 h (Fig. 5B; t = 3.369; df = 8, p = 0.0281). A significant increase in NCS-1 protein levels was also found in the frontal cortex of mice treated with CORT for 7 weeks (Fig. 5C; t = 6.145, df = 10, p = 0.0036). Our data suggest that the intracellular concentrations of Ca^2+^ are regulated by CORT, and increased Ca^2+^ may be involved in the downregulation of Flk1 by CORT.

### Long-term Continuous CORT Treatment Decreases Serum CORT Levels

CORT levels were analysed in the serum samples collected from mice treated with vehicle or CORT for 7 weeks. We found a significant reduction in serum CORT levels in CORT-treated mice [113.2±14.43 ng/mL vs 45.25±9.78 ng/mL (mean±SE); t = 3.659, df = 8, p = 0.008].

### GR Downregulation Is Involved in Long-term Continuous CORT-induced Downregulation of Flk1

**Figure 6:**
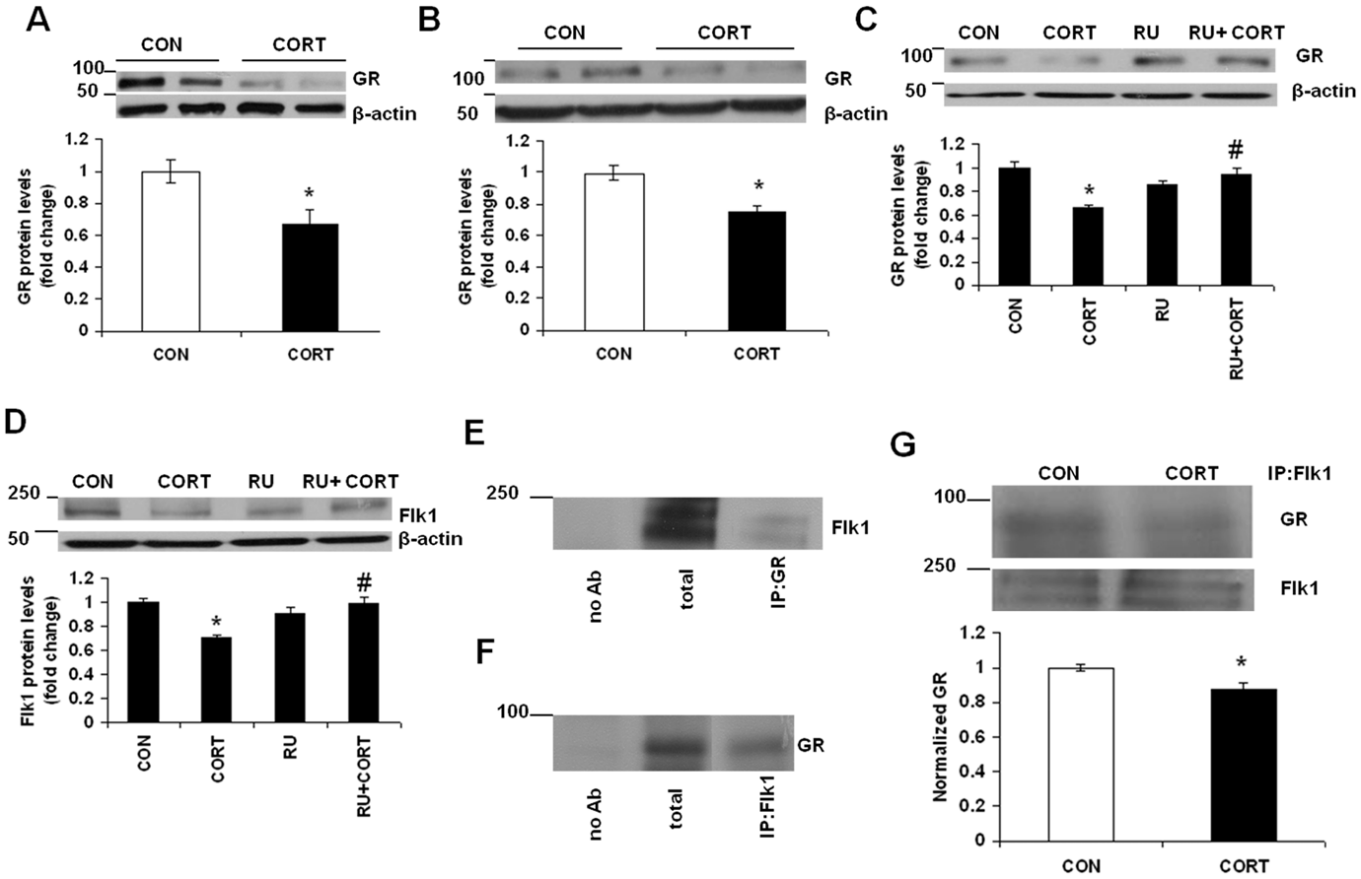
GR downregulation is involved in chronic CORT-induced downregulation of Flk1. (A) GR downregulation following chronic CORT exposure in neurons. CORT (CORT; 1 µM) was applied to mouse primary cortical neurons at DIV 5. GR protein levels were determined by western blotting analysis at 48 h following CORT treatment. CON means DMSO treatment. Data represent mean±SE (*n* = 5) expressed as fold change in GR protein levels as compared to CON. *P<0.05 (t test). (B) Chronic CORT treatment increases GR protein levels in mouse frontal cortex. GR protein levels in frontal cortex of mice treated with CORT (5 mg/kg) or vehicle control (CON; 0.45% hydroxypropyl-β-cyclodextrin) for 7 weeks were determined by western blot analysis. Data represent mean±SE (*n* = 5) expressed as fold change in GR protein levels as compared to CON. β-actin is the loading control.*P<0.05 (*t* test). (C) RU486 (RU, a GR antagonist) blocked CORT-induced reduction in GR protein levels. RU (1 µM) was applied 30 min before CORT (1 µM) treatment to cultured neurons at DIV5. Cell lysates were collected at 48 h after CORT treatment and GR protein levels were determined by western blot analysis. CON means DMSO treatment. Data represent mean±SE (*n* = 5) expressed as fold change in GR protein levels as compared to CON. *P<0.01 versus CON; #*P*<0.01 versus CORT (Bonferroni's test). (D) RU486 (RU, a GR antagonist) blocked CORT-induced reduction in Flk1 protein levels. Data represent mean±SE (*n* = 5) expressed as fold change in Flk1 protein levels as compared to CON. *P<0.01 versus CON; #*P*<0.01 versus CORT (Bonferroni's test). (E) Western blot analysis of Flk1 protein expression after immunoprecipitation with GR antibody in lysates collected from DIV6 neurons. NoAb: no anti-GR antibody; total: 10% input from total cell lysates. (F) Western blot analysis of GR protein expression after immunoprecipitation with Flk1 antibody in lysates collected from DIV6 neurons. NoAb: no anti-Flk1 antibody. (G) Immunoprecipitation of Flk1 in cell lysates from corticosterone (CORT) or vehicle control (CON; DMSO) treated for 48 h. Western blotting was performed with anti-GR and anti-Flk1 antibodies. Data represent mean±SE (n = 4) expressed as fold change in GR protein levels (normalized to Flk1 protein levels) as compared to CON. *P<0.05 versus CON (*t* test).

We examined the possible role of GR in chronic CORT-induced Flk1 downregulation. We found a significant reduction in GR protein levels at 48 h following CORT treatment in cortical neurons (Fig. 6*A*; t = 2.981, df = 8, p = 0.04). A significant reduction in GR was also observed in frontal cortex of mice treated with CORT for 7 weeks (Fig. 6*B*; t = 4.05, df = 8, p = 0.015). Pretreatment with RU486 (a GR antagonist) prevented GR downregulation by CORT in neurons (Fig. 6*C*; F(3, 16)  = 12.70, p<0.01). In addition, CORT-induced reduction in Flk1 was not observed when neurons were treated with CORT and RU486 (Fig. 6*D* *;* F(3, 16)  = 8.616, p<0.05). These results suggest that the downregulation of Flk1 following chronic CORT exposure is mediated through GR. Since we found a significant reduction in GR following CORT exposure, we examined the possible interaction between GR and Flk1 in neurons. We found coprecipitated Flk1 following immunoprecipitation with anti-GR antibody (Fig. 6*E*). Next, we examined coprecipitated GR after immunoprecipitation with anti-Flk1 antibody. We found significant coprecipitated GR in cortical neurons following immunoprecipitation with anti-Flk1 antibody (Fig. 6*F*). Chronic CORT treatment significantly reduced the coprecipitated GR as compared to vehicle treatment (Fig. 6G; t = 2.92, df = 6, p = 0.038).

### Reduced Flk1 and GR Protein Levels in Prefrontal Cortex of Schizophrenia Subjects

**Figure 7:**
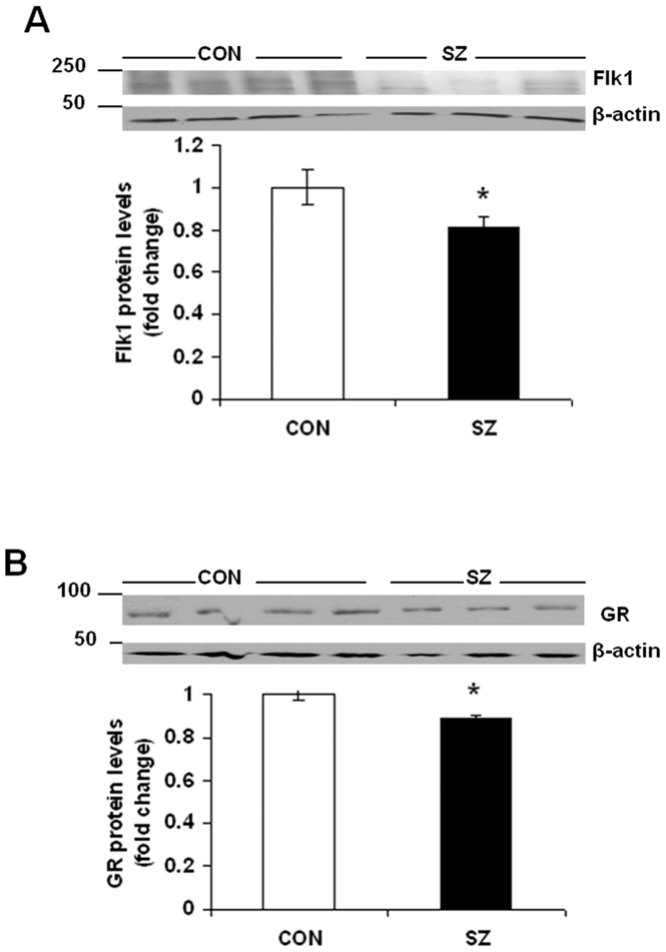
Reduced Flk1 and GR protein levels in prefrontal cortex of schizophrenia subjects. (*A*) Reduced Flk1 protein levels in prefrontal cortex samples from schizophrenia subjects. Flk1 protein levels in the prefrontal cortex of schizophrenia (SZ; n = 10) and control (CON; n = 8) subjects were determined by western blot analysis. Data represent mean±SE expressed as fold change in Flk1 protein levels as compared to CON. *P<0.05 versus CON (*t* test). (*B*) Reduced GR protein levels in prefrontal cortex samples from schizophrenia subjects (SZ; n = 10) as compared to control subjects (CON; n = 8). Data represent mean±SE expressed as fold change in GR protein levels as compared to CON. *P<0.05 versus CON (*t* test).

Studies were also carried out using postmortem prefrontal cortex samples from schizophrenia and control subjects. Western blot analysis showed a significant reduction in Flk1 protein levels in prefrontal cortex of schizophrenia subjects as compared to controls (Fig. 7*A*; t = 2.282, df = 16, p = 0.0365). In addition, we found a significant reduction in GR protein levels in samples from schizophrenia subjects (Fig. 7*B*; t = 3.795, df = 16, p = 0.001). Collectively, these results extend our findings on Flk1-GR interaction in cortical neurons following CORT exposure.

### CORT Treatment Did Not Change Body Weight and Water Intake in Mice

There were no differences in relative body weight gain during the experiment or water intake in mice treated with vehicle or CORT (data not shown).

## Discussion

Our data report the inhibitory effects of long-term continuous CORT treatment on Flk1 expression in mouse frontal cortex. Chronic stress and exogenous glucocorticoid exposure are known to result in neurochemical and behavioral abnormalities in rodents. Our studies have used 1 µM CORT in the in vitro studies and the above concentration has been shown to produce neuroprotective effects when the neurons are exposed to CORT for shorter time periods such as 5 to 15 min [1]. Although acute CORT treatment was found to be neuroprotective, the chronic treatment of CORT has been shown to cause adverse effects in central nervous system [7]. The dose and duration of CORT used (5 g/kg) in our in vivo study has previously been shown to cause anxiety and depression-like behavior in mice [5]. Our studies show that long-term continuous CORT exposure dramatically reduces Flk1 protein levels in cortical neurons in vitro, and frontal cortex and serum in vivo. Although we did not find any neuronal cell death even at 72 h following CORT exposure, the changes in Flk1 protein levels observed in our study may have a direct impact on the neuronal cell proliferation. It is well known that Flk1 plays an important role in neurogenesis [8]. A recent study has reported inhibition of neurogenesis following 5 mg/kg CORT administration for 7 weeks [5]. In the present study, we found a decrease in serum CORT levels in mice treated with CORT for 7 weeks. It is known that the circulating glucocorticoids exert ‘feedback’ to the HPA axis to turn off the glucocorticoid secretion and maintain the right range of glucocorticoids [39]. In addition, exogenous CORT treatment has been shown to flatten the diurnal CORT rhythm rather than an absolute increase in circulating CORT levels [40]. In the present study, the blood samples were collected between 3 p.m. and 4 p.m. It has been reported that CORT treatment inhibits the normal p.m. rise in peripheral CORT levels in rodents, whereas control animals demonstrate a normal circadian rhythm of CORT [41].

Because CORT significantly decreased Flk1 protein levels in cortex, we investigated the effects of CORT on signaling proteins downstream of Flk-1. The binding of VEGF to its tyrosine kinase receptors induces dimerization and autophosphorylation of the tyrosine residues [42]. Two high affinity tyrosine kinase receptors of VEGF, Flt-1 and KDR/Flk-1, are expressed on the cell membrane of neurons [10]. Most of the VEGF effects on neuroplasticity including neurogenesis and cognition are mediated by interaction with KDR/Flk-1 [11]. As the major downstream target of KDR/Flk-1, Akt signaling pathway is an important mediator of neuroplasticity [14]. Following treatment with CORT, a reduction in phosphoAkt (ser273) levels was found in cortical neurons. The changes in Akt signaling following CORT treatment was further confirmed by a significant reduction in phospho-mTOR levels, one of Akt's downstream effector molecules. We also found a significant increase in phospho-PTEN levels, an upstream regulator of PI3K/Akt signaling. PTEN is one of the most studied regulators of Akt activity. PTEN regulates the PI3K/Akt signaling by controlling the levels of phosphatidylinositol-3,4,5-trisphosphate (PIP3). The balance between PI3K and PTEN activity determines the intracellular levels of PIP3 and downstream activity of Akt. Activation of PTEN has been shown to result in the inhibition of Akt and other downstream targets including mTOR. This serine–threonine-kinase member of the PI3K-family recently gained major interest as therapeutic target due to its key regulatory role in various cellular functions [43]. Thus, long-term continuous CORT treatment inhibits a complex pathway comprised of PI3K, Akt and mTOR kinases, which regulates a number of cellular functions in CNS [44]. Interestingly, a recent study has shown that the blockade of mTOR signaling completely blocked ketamine induction of synaptogenesis and behavioral responses in animal models of depression [45].

Since we found a significant inhibitory effect of CORT on Flk1 signaling in neurons, we next examined the mechanisms of Flk1 regulation under long-term continuous CORT exposure. Our studies on VEGF protein levels found a significant increase in VEGF protein levels both in vivo and in vitro following CORT exposure in frontal cortex. However, we found a significant reduction in serum VEGF protein levels in mice treated with CORT for 7 weeks. These data suggest that a feedback mechanism may operate to increase VEGF production in cortex in response to inhibition of the VEGF signaling pathway. However, the possibility of increase in VEGF in cortex as a response to reduction in peripheral VEGF protein levels following CORT treatment can not be ruled out. Such an increase in VEGF in cortex could result in inhibition of Flk1 signaling and reduction in Flk1 levels in periphery. But, further studies are warranted to understand the above possible mechanisms. It is important to note that PI3K signaling also plays an important role on VEGF regulation [46]. To determine whether the changes observed in PI3K pathway following CORT exposure is upstream to VEGF we conducted studies using specific inhibitors of the above pathway in cortical neurons. Our results did not show any significant effect of PI3K inhibitor on VEGF protein levels in CORT-treated cells. These results suggest that PI3K signaling may not be involved in the regulation of VEGF expression under CORT exposure.

Ca^2+^ is an important intracellular messenger in neurons, regulating a variety of neuronal processes such as neurotransmission and signal transduction. At the cellular level, glucocorticoid-induced elevations in [Ca^2+^] result in changes in synaptic plasticity and neuronal excitability [47], [48]. NCS-1 is one of the members of a large family of EF-hand Ca^2+^-binding proteins, which may act as Ca^2+^ sensors or Ca^2+^ buffers in mediating the actions of Ca^2+^ [49]. The increased expression of NCS-1 protein as well as the inhibition of CORT-induced reduction in Flk1 protein levels by BAPTA-AM in our study suggests that Ca^2+^ may be in involved in mediating CORT effects on Flk1 expression. However, the caveat exists that the increased expression of NCS-1 may be in response to neuronal damages induced by CORT exposure. In accordance with this, we found little loss of cells in CORT-treated cortical neurons. This lack of cell death would be the result of the increased expression level of endogenous NCS-1 in the neurons. Because evidence indicates that chronic stress leads to upregulation of multiple antiapoptotic molecules as an adaptive response [50], it is possible that the neuronal insults induced by long-term continuous CORT exposure upregulates NCS-1 expression and promotes cellular survival in neurons. Interestingly, an up-regulation of NCS-1 has also been reported in the cortex of schizophrenic and bipolar patients, demonstrating the involvement of NCS-1 in neuropsychiatric disorders [51]. Although the underlying mechanism of changes in NCS-1 expression in schizophrenia and bipolar disorder is not known, it might be associated with the altered Ca^2+^ signaling reported in these disorders [52].

A reduction in GR-Flk1 interaction was found in cortical neurons following CORT exposure. The reduction in GR-Flk1 interaction might be as a result of the downregulation of GR protein levels by CORT. Chronic glucocorticoid has been shown to downregulate the interaction of GR with TrkB, another tyrosine kinase receptor in neurons [53]. The reduction in Flk1 protein levels following CORT was inhibited by GR antagonist, RU486. Flk1 might be part of a protein complex with GR, and the reduction in GR following CORT exposure might result in the dissociation of Flk1 from GR and Flk1 signaling is thus inhibited. Similar mechanisms have been suggested for GR-TrkB interaction in neurons [53] and GR-TCR interaction in T-cells [54]. Together, our data suggest the possible role of GR in mediating CORT effects on Flk1.

We found a significant reduction in Flk1 and GR protein levels in prefrontal cortex samples from schizophrenia subjects. Although the schizophrenia and control subjects in our sample set are not well matched in their demographic variables we did not find any significant difference between controls and schizophrenia subjects in any of the confounding variables, such age at death, PMI, brain weight, refrigeration interval, gender and duration of illness. In addition, no significant correlation was found between the protein levels of either GR or Flk1 levels and the above confounding variables (data not shown). However, we can not rule out the possible influence of pH on the above protein levels [55]. Unfortunately, the sample cohort used in the present study lack the information on pH.

**Figure 8:**
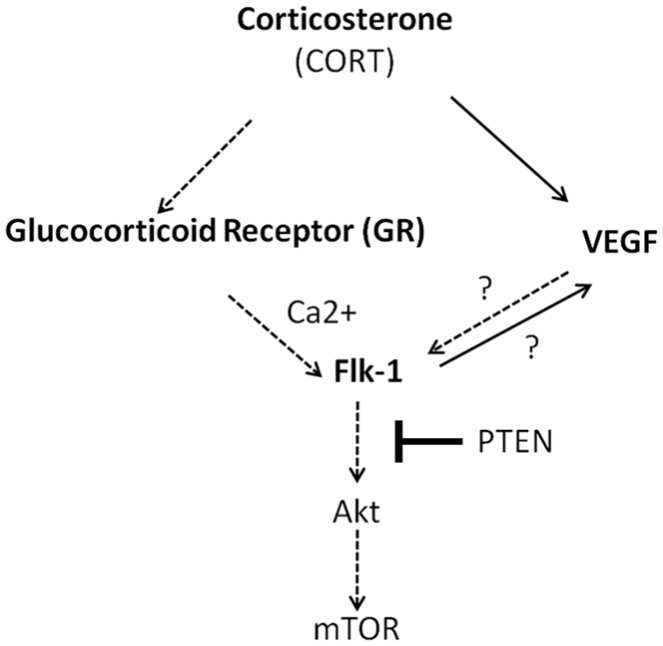
Proposed model showing the effects of corticosterone on VEGF/Flk1 signaling pathway in mouse frontal cortex. The signaling events induced by corticosterone (CORT) are mediated through the Glucocorticoid receptor (GR). The reduction in Flk1 levels following long-term continuous CORT exposure results in the activation of PTEN, but inhibition of Akt and mTOR phosphorylation. The effects of CORT on Flk1 are mediated through calcium (Ca^2+^). CORT exposure results in increased levels of VEGF in cortex. The role of VEGF in Flk1 regulation (or vice versa) under CORT exposure remains unknown. Solid arrows represent activation, whereas dashed arrows represent inhibition of the pathways.

In conclusion, this study demonstrates altered Flk1 expression in the frontal cortex in response to long-term continuous CORT treatment (Fig. 8). This could have relevance in understanding neurobiological effects of glucocorticoids and of chronic stress.

## Materials and Methods

### Animals

All in vivo experiments were conducted in adult male CD1 mice (Charles River Laboratories, Wilmington, MA, USA). All CORT-treated mice were 7–8 weeks old and weighed 25–30 g at the beginning of the treatment. Mice were maintained on a 12-h light–dark cycle with the lights on at 0700 hours, and were housed four per cage. Food and water were provided *ad libitum*. All in vitro experiments were done in cerebral cortical neuronal cultures from embryonic day 16 mouse fetuses. Animal use procedures were performed after being reviewed and approved by Medical College of Georgia, Committee on Animal Use for Research and Veterans Affairs Medical Center Subcommittee on Animal Use. Procedures were consistent with the Association for Assessment and Accreditation of Laboratory Animal Care (AAALAC) guidelines as per Public Health Service Policy on Humane Care and Use of Laboratory Animals.

### Tissue sample preparation

At the end of 7 week-treatment, mice were killed by decapitation, and the frontal cortex from experimental and control mice were separately dissected. The dissection level for the frontal cortex, 2.34 mm anterior to bregma, was chosen according to the mouse brain stereotaxic coordinates [26]. The tissue samples were homogenized in radioimmune precipitation assay (RIPA) buffer (10 mM Tris-HCl, pH 7.5, 150 mM NaCl, 0.1% sodium dodecyl sulfate (SDS), 1% Nonidet P-40, and 1% sodium deoxycholate) for western blotting. RIPA buffer enables efficient cell lysis and protein solubilization while avoiding protein degradation and interference with immunoreactivity. This buffer was supplemented with a protease inhibitor cocktail (Sigma) containing 104 mM 4-(2-Aminoethyl) benzenesulfonyl fluoride hydrochloride (AEBSF), 0.08 mM aprotinin, 2 mM leupeptin, 4 mM bestatin, 1.5 mM pepstatin A and 1.4 mM E-64. After 15 min incubation on ice, the extracts were clarified by centrifugation at 15,000 g for 15 min at 4°C and stored at −70°C. Protein concentration was determined by the bicinchoninic acid method (BCA Protein Assay Kit, Sigma).

### Cerebral Cortical Neuronal Cultures

Mouse cortical neurons were cultured as described previously [26]. Briefly, cerebral cortices from CD-1 murine embryos (E16) were aseptically dissected and plated at 3.5 × 10^5^ cells per well on polyethyleneimine-coated 6-well plates. Neurons were cultured in Neurobasal medium supplemented with B27, 2 mM L-glutamine, and antibiotics (Invitrogen). On the third day *in vitro* (DIV3), media was replaced with Neurobasal supplemented with B27 minus antioxidants, glutamine, and antibiotics. Purified neuronal cultures were routinely >97% neurons, as assessed by MAP-2 immunostaining. Neurons were used for treatments between DIV 5 and 7. Following treatments in culture, cells were washed in Phosphate Buffered Saline (PBS) and collected in ice-cold RIPA buffer. Protein concentration was determined by the BCA method.

### Drug Treatment

#### In vivo studies

CORT (4-pregnen-11b-diol-3 20-dione 21-hemisuccinate; Sigma, St Louis, MO, USA) was dissolved in vehicle (0.45% hydroxypropyl-β-cyclodextrin, Sigma, St Louis, MO, USA). CORT (35 µg/mL, equivalent to 5 mg/kg/day) was delivered *ad libitum* in the drinking water. The dose and duration of CORT treatment in mice were selected based on an earlier study [5] where the above dose and duration of treatment with CORT induced anxiety and depression-like behaviors in mice. Control mice received 0.45% hydroxypropyl-β-cyclodextrin as vehicle.

#### In vitro studies

Primary cortical neurons were treated with CORT (1 µM) or vehicle (DMSO). CORT concentration for in vitro study was selected based on an earlier study [1] where acute exposure with above dose was found neuroprotective in primary cortical neurons. The treatment was carried out with a single application of CORT or vehicle in 48 h treatment group whereas the solutions were replenished after 48 h in the 72 h treatment groups. Neuronal cell viability was assessed at 48, 72 and 96 h following CORT exposure and Flk1 expression was examined at 12, 24, 48 and 72 h after CORT treatment. The analyses of other proteins were carried out at 48 and/or 72 h following CORT exposure.

### Determination of neuronal cell viability in culture

Neuronal cell viability in culture was assessed using the 3-(4,5-dimethylthiazol-2-yl)-2,5-diphenyltetrazolium bromide (MTT) reduction assay [27]. Briefly, following treatments, 3-(4,5-dimethylthiazol-2-yl)-2,5-diphenyltetrazolium bromide [5 mg/mL in phenol-red free Roswell Park Memorial Institute medium (RPMI)-1640] was added to each well for 4 h at 37°C. Following incubation, formazan salts were dissolved in acidic isopropanol and absorbance was measured using a VERSAmax microplate reader (Molecular Devices, Sunnyvale, CA, USA) at 540 nm using a reference wavelength of 690 nm. All readings were compared with the control treatment group.

### Western blot analysis

Equal amounts of protein were resolved in SDS–polyacrylamide gels and transferred electrophoretically onto a nitrocellulose membrane. The membrane was blocked for 1 h in PBS solution with the detergent Tween 20 (PBST**;** 3.2 mM Na2HPO4, 0.5 mM KH2PO4, 1.3 mM KCl, 135 mM NaCl, 0.05% Tween 20, pH 7.4.**)** and 5% non-fat milk or 5% BSA. The membranes were incubated overnight with the indicated primary antibodies. The primary antibodies used were anti-phospho- Akt, anti-Akt, anti-Flk-1, anti-phospho-mTOR, anti-mTOR, anti-phospho-PTEN, anti-PTEN (all antibodies were from Cell signaling, Beverly, MA, USA), anti-GR, anti-NCS-1 (both were from Santa Cruz Biotech, CA, USA) and anti-β-actin (Sigma, St Louis, MO, USA). The membranes were washed again with PBST then incubated with secondary antibody for 1 h. Proteins were visualized by enhanced chemiluminescence. The films were subsequently scanned, and band intensity was quantified by densitometry software (Image J, NIH). The western blot data for Flk1, GR and NCS-1 were corrected for corresponding β-actin values. The data for phosphorylated forms of proteins were corrected for corresponding total protein values. For VEGF, tissue or cell lysates were subjected to heparin beads (Sigma-Aldrich) as described previously [28]. In brief, the beads were pelleted at 5000 *g* for 1 min, washed in 400 mM NaCl and 20 mM Tris, and loaded onto a 4 to 20% gradient Tris glycine precast gel (Bio-Rad Laboratories). After blocking, the membrane was incubated with VEGF primary antibody (Calbiochem, Gibbstown, NJ, USA). The band was visualized and quantified as described above.

### Serum VEGF assay

Quantification of serum VEGF was performed with a DuoSet enzyme-linked immunosorbent assay (ELISA) (R&D Systems, Minneapolis, IN, USA) according to the manufacturer's specifications.

### Serum CORT assay

At the end of 7-week treatment, mice were killed by decapitation (1500–1600 h). Trunk blood was harvested and serum collected for CORT analyses. Serum was assayed for CORT using a quantitative competitive enzyme immunoassay kit (Cat # KA0468 V.04) from Abnova, Taipei according to manufacturer's instructions. Intra-assay and inter-assay measures of variability were 4.8% and 7.3% respectively. Serum concentrations were determined from a software generated standard curve and reported in ng/mL.

### Serum Flk1 assay

Serum samples were collected as described above and soluble Flk1 protein levels were determined with a quantitative sandwich enzyme immunoassay kit (#MVR200B; R&D Systems) according to the manufacturer's specifications.

### Human prefrontal cortex samples

The prefrontal cortex samples from schizophrenia and control subjects were obtained from the Human Brain and Spinal Fluid Resource Center (Los Angeles, California, USA). A description on the demographic details is given in Table 1. No significant difference was found between controls and schizophrenia subjects in any of the confounding variables, such age at death, PMI, brain weight, refrigeration interval, gender and duration of illness. The samples were shipped frozen and stored at − 80°C until analysis. Grey matter was removed from a 1.5–2.0 cm thick coronal slab of the frontal cortex anterior to the corpus callosum and the prefrontal cortex was dissected [29]. Prefrontal cortex tissue was homogenized in a homogenizing buffer containing 20 mM Tris–HCl (pH 7.4), 2 mM EGTA, 5 mM EDTA, 1.5 mM pepstatin, 2 mM leupeptin, 0.5 mM phenylmethylsulfonyl fluoride, 0.2 U/mL aprotinin, and 2 mM dithiothreitol, using a Polytron homogenizer. The homogenate was centrifuged at 15,000 rpm for 15 min at 4°C. Protein concentration in the supernatant was determined with BCA Reagent. Flk1 and GR protein levels were determined by western blot analysis as described above using β-actin as a loading control [30].

**Table 1:** Demographic data for postmortem samples.

	Control	Schizophrenia
Age (years, mean ± SE)	72.50±2.26	69.00±4.44
PMI (years, mean ± SE)	11.89±0.58	17.05±2.12
Gender (M/F)	7/1	5/5
Brain Weight (g, mean ± SE)	1225.00±40.17	1090.56±72.58
Duration of Illness (years, mean ± SE)	0	37.86±6.44
Refrigeration Interval (h, mean ± SE)	1.37±0.18	2.15±0.38

### Statistical Analysis

Data are expressed as mean±SE. Two-way ANOVA followed by post hoc one-way ANOVA with Bonferroni's multiple comparison test was used in analyzing data from time dependent studies. Comparisons between multiple groups were done by one-way ANOVA. Individual comparisons between two groups were performed with Student's t test. Probability (*P*) values of less than 5% were considered significant.
